# Efficacy and safety of stem cell therapy for dry eye syndrome in Sjögren’s syndrome: a systematic review and meta-analysis

**DOI:** 10.3389/fimmu.2026.1834453

**Published:** 2026-04-29

**Authors:** Jizhong Zhang, Wenshuang Wang, Changyong Li

**Affiliations:** Institute of Rehabilitation Medicine, Qilu Medical University, Zibo, Shandong, China

**Keywords:** dry eye syndrome, meta-analysis, Sjögren’s syndrome, stem cells, systematic review

## Abstract

**Background:**

Stem cell therapy holds considerable potential for treating dry eye syndrome, though it has yet to receive clinical approval.

**Aim:**

This paper systematically quantifies the efficacy and safety of stem cell-based therapies in dry eye syndrome caused by Sjögren’s syndrome, aiming to provide information for dry eye treatment efforts.

**Methods:**

This systematic review collected literature published prior to 16 January 2026 in PubMed, Embase, and the Cochrane Library concerning stem cell therapy for Sjögren’s syndrome-induced dry eye disease. Efficacy outcomes comprised: OSDI scores, along with changes in NIKBUT first, Oxford score, and Schirmer test results. Safety outcomes comprised commonly reported adverse events. Continuous outcomes were expressed as mean difference (MD), while dichotomous outcomes used single-group rates, both presented with 95% CI. All data analyses were conducted using Review Manager 5.4 software, adhering to the PRISMA guidelines.

**Results:**

This meta-analysis included five studies involving 114 patients with dry eye syndrome. Results demonstrated that stem cell therapy significantly altered the OSDI score compared to pre-treatment levels: -15.10 (95% CI: -18.65, -11.56; P < 0.00001). NIKBUT first scores increased significantly by 3.26 points post-treatment (95% CI: 2.17, 4.34; P < 0.00001). The Oxford score showed a change of -0.20 (95% CI: -0.85, 0.45; P = 0.55) post-treatment. The Schirmer test score exhibited an overall change of 3.87 (95% CI: 1.93, 5.81; P < 0.0001). Specifically, the MD at 2 weeks, 4 months, and 12 months was 8.76 (95% CI: 0.58, 16.94; P < 0.04), 3.52 (95% CI: 1.66, 5.38; P < 0.002), and 5.10 (95% CI: 0.24, 9.96; P < 0.04), respectively. The incidence of injection pain at 4 weeks was 14% (95% CI: -11%, 39%, P = 0.28). Ocular discomfort occurred in 16% of subjects at 4 weeks (95% CI: -4%, 35%, P = 0.11). Periorbital oedema occurred in 14% of subjects at 4 weeks (95% CI: -11%, 39%, P = 0.28). Blurred vision and periorbital paresthesia occurred at treatment with rates of 21% (95% CI: -7%, 50%, P = 0.14) and 15% (95% CI: -4%, 35%, P = 0.12), respectively.

**Conclusion:**

This meta-analysis indicates that stem cell therapy effectively reduces OSDI scores, increase Schirmer test scores, and enhances tear film stability in dry eye syndrome caused by Sjögren’s syndrome. However, the increased incidence of adverse events in the short term suggests that benefits and risks must be carefully weighed prior to clinical application, with rigorous monitoring and close follow-up required throughout treatment.

**Systematic review registration:**

https://www.crd.york.ac.uk/PROSPERO/view/CRD420261287054, identifier CRD420261287054.

## Introduction

Sjögren’s syndrome (SS) is a chronic autoimmune disorder affecting millions worldwide. Dry eye disease (DED) is a multifactorial condition, with factors such as meibomian gland dysfunction, infection, and medication all contributing to its development (1, 2). Its primary manifestations include tear film instability, hyperosmolarity, and ocular surface inflammation, leading to symptoms such as ocular discomfort and pain. SS-associated dry eye represents the most prevalent and severe form of DED (3, 4).

DED can be categorised into two primary types: evaporative dry eye (EDE) and aqueous deficiency dry eye (ADDE), though patients often present with both simultaneously. Due to its complex aetiology and the poor correlation between clinical signs and symptoms, the clinical diagnosis of DED is challenging. This lack of correlation frequently leads to misdiagnosis and inadequate treatment of DED (5). Current primary treatments for SS-DED encompass multiple approaches, including topical and oral medications such as artificial tears, immunosuppressants, and glucocorticoids, alongside surgical interventions and adjunctive physical therapies (1). However, these treatments yield limited efficacy in severe cases and carry risks of long-term adverse effects.

Stem cells possess unlimited differentiation potential, capable of differentiating into multiple phenotypes. Represented by mesenchymal stem cells (MSCs), they secrete abundant immunomodulatory factors (6–9). Stem cells have demonstrated anti-inflammatory and immunoregulatory capabilities, alleviating systemic inflammation without inducing severe adverse reactions. Consequently, they represent an ideal research subject for inflammatory diseases such as SS-induced ADDE (10, 11). Adipose-derived mesenchymal stem cells (AT-MSCs) can be obtained via minimally invasive liposuction, offering favourable patient compliance. Umbilical cords are typically discarded post-delivery, enabling MSC acquisition under ethically compliant conditions.

Numerous preclinical studies across diverse animal models have explored the therapeutic potential of MSCs for ADDE (12, 35). Clinical trials have progressively commenced in recent years, though their comprehensive efficacy remains unresolved. This review incorporates randomised controlled trials and open-label, self-controlled studies reporting stem cell therapies for dry eye syndrome. It systematically quantifies the efficacy and safety of stem cell treatments for dry eye syndrome associated with Sjögren’s syndrome, aiming to inform clinical practice.

## Methods

### Search strategy

This review collates literature published in PubMed, Embase, and the Cochrane Library databases up to 16 January 2026 concerning stem cell therapy for dry eye syndrome caused by Sjögren’s syndrome. Core search terms included: stem cells, Sjögren’s syndrome, and dry eye syndrome. The specific search strategy is detailed in (**Supplementary File**). This systematic review was registered in the Centre for the Evaluation and Dissemination of Health Research and Innovation’s (CERDI) prospective systematic review registry, established in collaboration with the UK National Institute for Health Research (NIHR), under registration number CRD420261287054 (13). The systematic review was rigorously conducted and reported in accordance with the PRISMA guidelines.

### Inclusion criteria

P: Individuals with dry eye syndrome caused by Sjögren’s syndrome, with no restrictions on age, gender, or ethnicity. I: Intervention comprising stem cell therapy from sources such as mesenchymal stem cells. C: Placebo or other treatments. O: Primary outcome: OSDI score; secondary outcomes: NIKBUT First, Oxford score, Schirmer test score. Adverse events: ocular discomfort, injection site pain, periorbital oedema, periorbital sensory disturbance, and blurred vision. Minimum follow-up duration: 3 months. S: Randomised controlled trial (double-blind or triple-blind), self-controlled clinical trial.

### Exclusion criteria

Dry eye syndrome not attributable to Sjögren’s syndrome will be excluded, along with other types of dry eye patient populations. Individuals who have previously undergone treatment of the lacrimal glands (LG) with autologous stem cells (ASCs) or other stem cell products will be excluded. Follow-up periods of less than three months will be excluded.

### Data extraction

**Figure 1:**
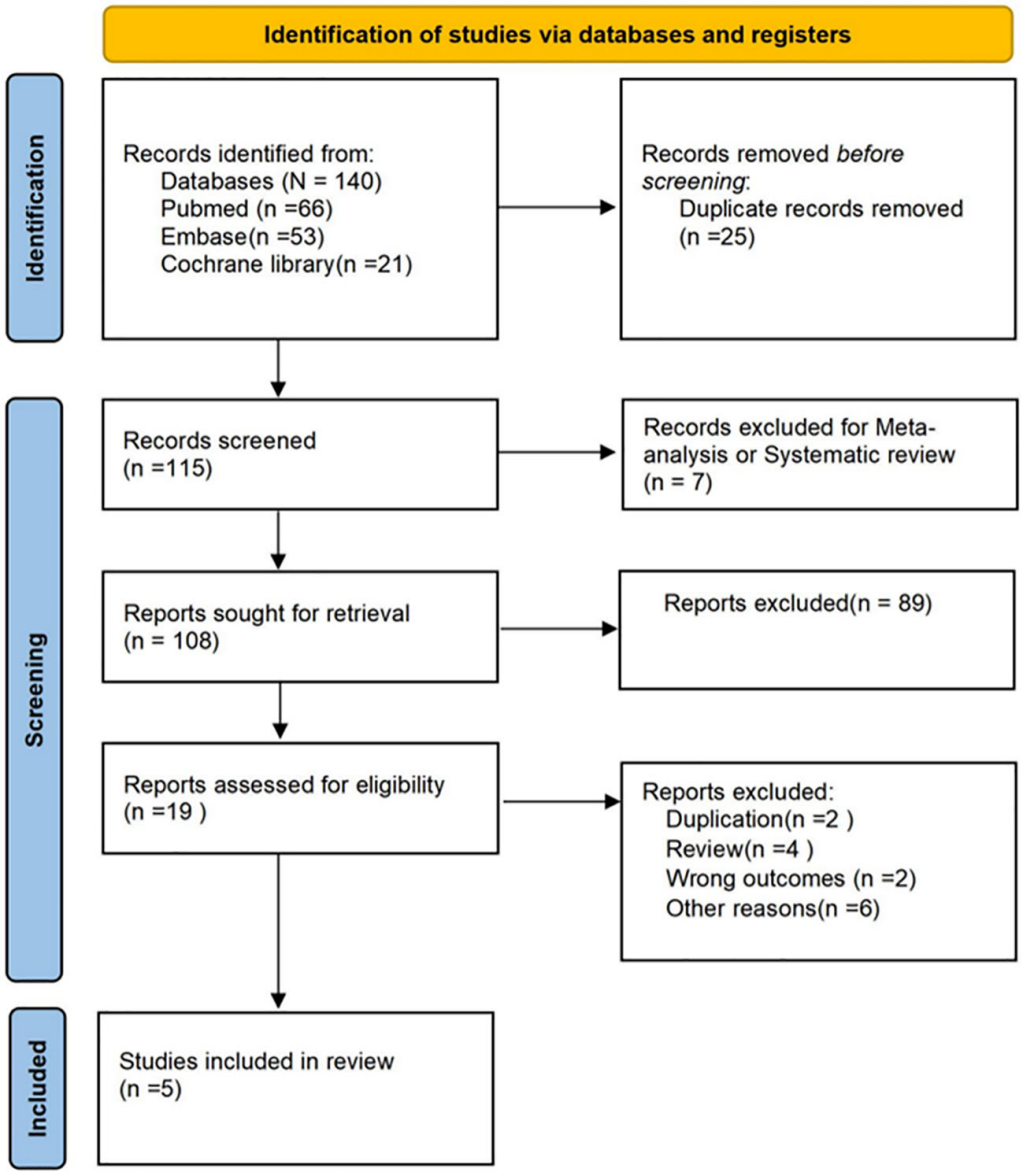
PRISMA literature screening flowchart process.

Two researchers (L.C.Y and W.W.S) will independently review the abstracts. Any discrepancies will be resolved by a third researcher (J.Z.Z). Should disagreements remain unresolved, the authors will be contacted. The specific retrieval process followed the PRISMA flow diagram (**Figure 1**). Research data extraction results are summarised in (**Table 1**), with patient characteristics (including demographic information and baseline features) included and excluded across different studies presented in tabular form. Standardised data sheets were used to collect the following information: 1. First author’s name and year; 2. Country; 3. Study design; 4. Study population (n); 5. Median age; 6. OSDI score; 7. NIKBUT first; 8. Oxford score; 9. Schirmer test score; 10. Intervention; 11. Stem cell sources.

**Table 1:** Characteristics of included studies and baseline characteristics of participants in included studies.

Author, year	Country	Study design	Population (N)	Age	OSDI	NIKBUT first	Oxford score	Schirmer test score	Intervetion	Stem cell sources
Michael Møller-Hansen 2024 (16)	Denmark	Double-blind randomised clinical trial	Sjögren’s Syndrome (SS)Associated Dry Eye (N = 54)	59(16.30)	39.80(12.22)	3.50(2.30)	2.00 (1.48)	2.50 (2.96)	Inject 3.96x10^6^ASCs(0.18ml)	Allogeneic adipose-derived stem cells
Azam Habibi2025 (17)	Iran	Phase I and II triple-blind, randomised controlled trials	Sjögren’s syndrome-associated dry eye (N = 16)	43.6(9.15)	34.00(6.81)	4.40 (1.70)	1.38 (0.92)	1.50 (4.44)	20μg/d, 14d	Umbilical cord MSCs
Di Zhang 2025 (18)	China	Open-label, prospective, single-arm, self-controlled clinical trial	Refractory dry eye disease (N = 31)	43(15.9)	40.67(27.94)	5.47(1.91)	1.11(1.31)	4.11 (2.68)	1x10^6^ MSCs/d, 14d	Umbilical cord MSCs
]Michael Møller-Hansen 2021 (19)	Denmark	Open-label, self-controlled clinical trial	Aqueous deficiency dry eye (N = 7)	59.9(10.06)	58.90(20.60)	3.70(1.50)	1.30 (1.00)	4.60 (0.70)	2.2x10^6^ ASCs(N = 4) 4.4x10^6^ ASCs(N = 3)	Allogeneic adipose-derived stem cells
Mojtaba Mohammadpour2025 (20)	Iran	Prospective self-controlled clinical trial	Sjögren’s Syndrome (SS)Associated dry eye (N = 6)	56.1(7.2)	48.61(8.40)	3.33(1.03)	1.67(0.52)	4.17(0.75)	22x10^6^ ASCs	Autologous adipose-derived stem cells

### Outcomes measures

Primary efficacy endpoint: OSDI score. Secondary efficacy endpoints: NIKBUT First, Oxford score, Schirmer test score. Safety endpoints: ocular discomfort, injection site pain, periorbital oedema, periorbital paraesthesia, and blurred vision. Follow-up duration was set at no less than 3 months. For post-treatment time points, we selected commonly reported follow-up intervals from the literature—2 weeks, 4 weeks, 4 months, and 12 months—to provide a more comprehensive overview. However, due to variations in follow-up durations across studies and data gaps, the number of included studies at each common time point was limited. Attempts to obtain unreported data from the authors (Michael Møller-Hansen et al.) were unsuccessful.

### Quality assesment

Two authors (L.C.Y and W.W.S) independently assessed risk of bias using the risk of bias assessment tool, with any disagreements resolved by a third author (J.Z.Z). For RCT studies, the Cochrane risk of bias tool RoB.2 was employed for assessment (14). For open-label, self-controlled clinical trials, the MINORS scale—a tool designed to evaluate the quality of non-randomised controlled trials—was utilised (15).

### Statistical analysis

Data transformation strictly adhered to the conversion protocol outlined in Chapter 6.5.2 of the Cochrane Handbook. Data extracted using Microsoft Excel were imported into Review Manager 5.4 for statistical analysis. Heterogeneity between trials was assessed using the I² test: I² < 25% indicated negligible heterogeneity, employing a fixed-effect model; I² > 50% (indicating substantial heterogeneity) necessitated a random-effects model. We explored sources of heterogeneity through subgroup analyses based on follow-up duration. Given the limited number of included studies, publication bias was not assessed.

Continuous outcomes were expressed as MD, while dichotomous outcomes used single-group rates, both presented with 95% CI. Differences were considered statistically significant (P < 0.05) and non-significant (P ≥ 0.05). Primary and secondary dry eye-related outcomes were compared against baseline values post-treatment. To address differences in study design, randomized controlled trials and self-controlled studies were synthesized using a harmonized analytical framework based on within-group change from baseline to post-treatment in stem cell-treated participants. Thus, treatment effects were not pooled as direct between-group comparisons, but rather as pre- versus post-treatment changes, which allowed results from different study designs to be combined on a common scale. Continuous outcomes were expressed as MD, while adverse events were summarized as single-group rates. Methodological quality was assessed using design-specific tools, with RoB 2 for randomized controlled trials and MINORS for self-controlled clinical studies. Commonly reported indicators across all literature were selected. Adverse event incidence rates were calculated. Similarly, for adverse events, commonly reported adverse events from the literature were selected. The occurrence of each adverse reaction at the endpoint was specifically detailed.

## Results

We retrieved a total of 140 studies. Through comprehensive search procedures and analytical methods, and following consultation among all authors, five studies were ultimately selected, encompassing 114 participants (16–20). The PRISMA flow diagram illustrates the complete screening process. For instance, in the Di Zhang 2025 study, the stem cell dose was 1 × 10^6^ MSCs/day, with a treatment course of 14 days. In the Mojtaba Mohammadpour 2025 study, the stem cell dose was 22 × 10^6^ ASCs administered as a single injection. Detailed demographic and baseline characteristics are presented in **Table 1**. Bias assessment revealed no apparent risk of bias (**Supplementary Figures 1, 2**; **Supplementary Table 1**).

**Figure 2:**
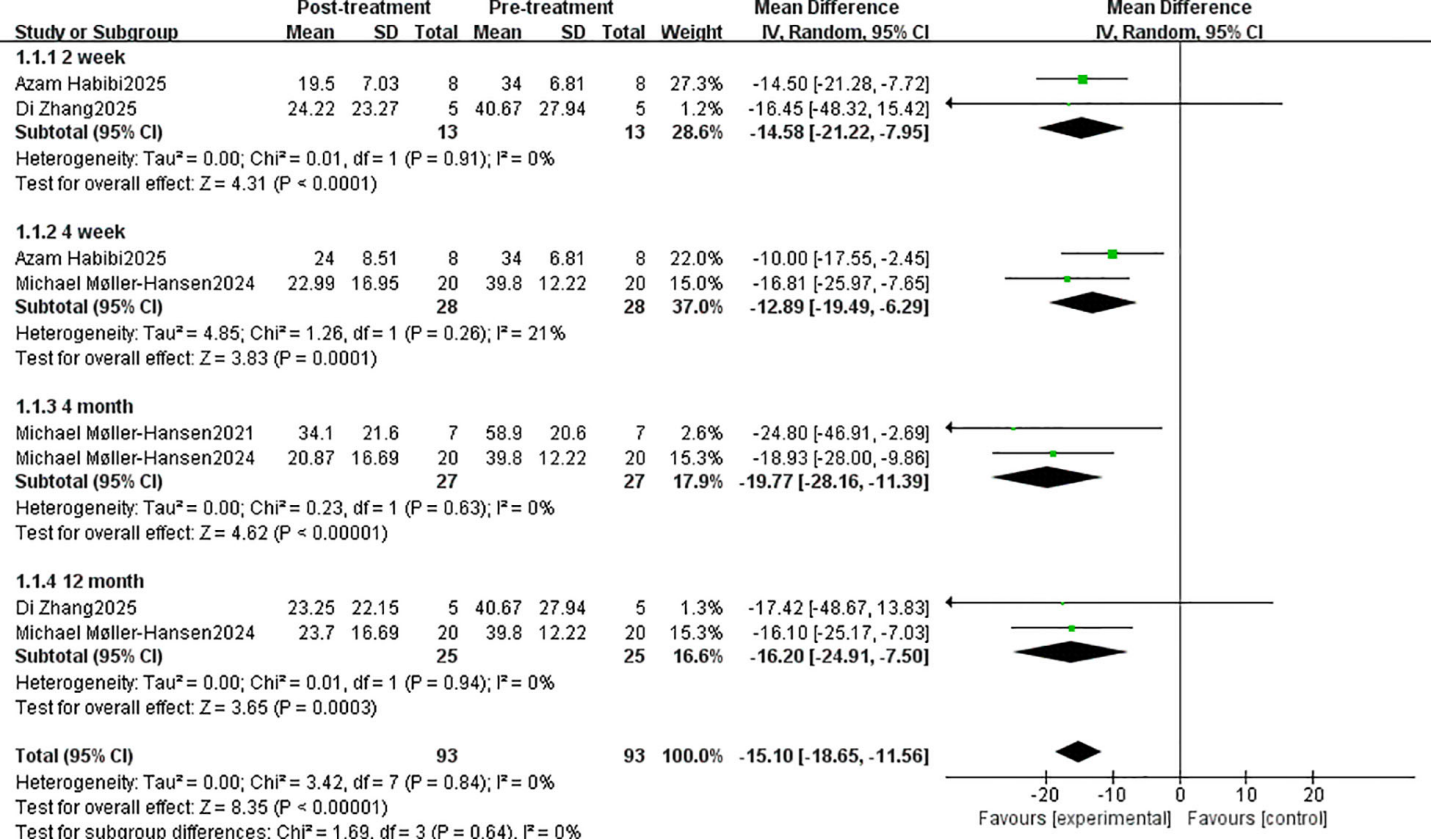
The OSDI score evaluates the efficacy of stem cell therapy.

The pooled analysis revealed that stem cell therapy significantly altered the OSDI score compared with pre-treatment levels: -15.10 (95% CI: -18.65 to -11.56, P < 0.00001). The MD at 2 weeks, 4 weeks, 4 months, and 12 months was -14.58 (95% CI: -21.22, -7.95; P < 0.0001), -12.89 (95% CI: -19.49, -6.29; P < 0.0001), -19.77 (95% CI: -28.16 to -11.39, P < 0.00001), and -16.20 (95% CI: -24.91 to -7.50, P < 0.0003) (**Figure 2**). Statistically significant differences were observed at all follow-up time points.

**Figure 3:**
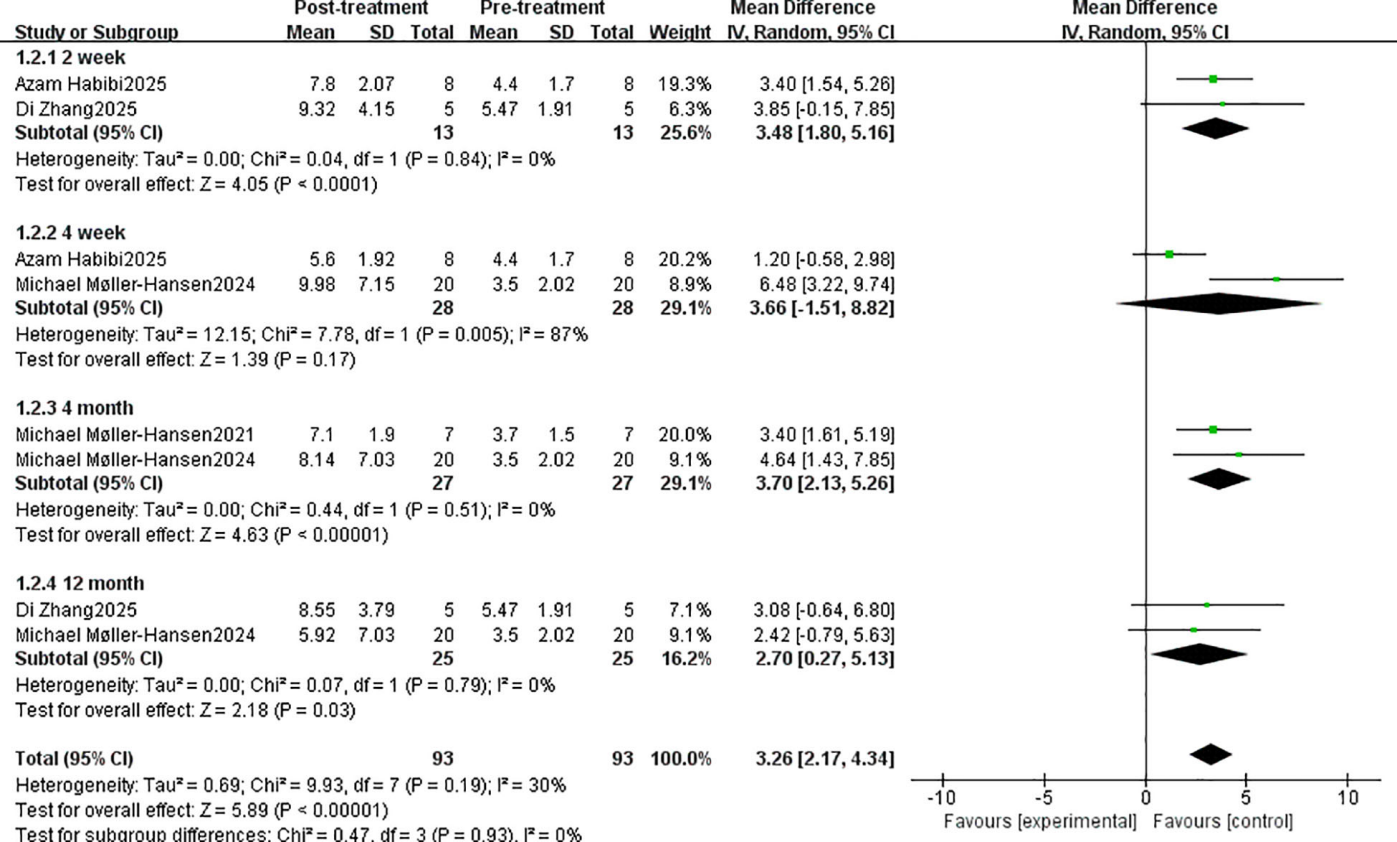
NIKBUT first scoring system evaluates the efficacy of stem cell therapy.

NIKBUT first treatment significantly increased by 3.26 (95% CI: 2.17, 4.34, P < 0.00001). The MD differences at 2 weeks, 4 weeks, 4 months, and 12 months were 3.48 (95% CI: 1.80, 5.16, P < 0.0001), 3.66 (95% CI: -1.51, 8.82, P = 0.17), 3.70 (95% CI: 2.13, 5.26, P < 0.00001), and 2.70 (95% CI: 0.27, 5.13, P < 0.03) at 2 weeks, 4 weeks, 4 months, and 12 months, respectively (**Figure 3**). Statistically significant differences were observed in all outcomes except at 4 weeks post-treatment.

**Figure 4:**
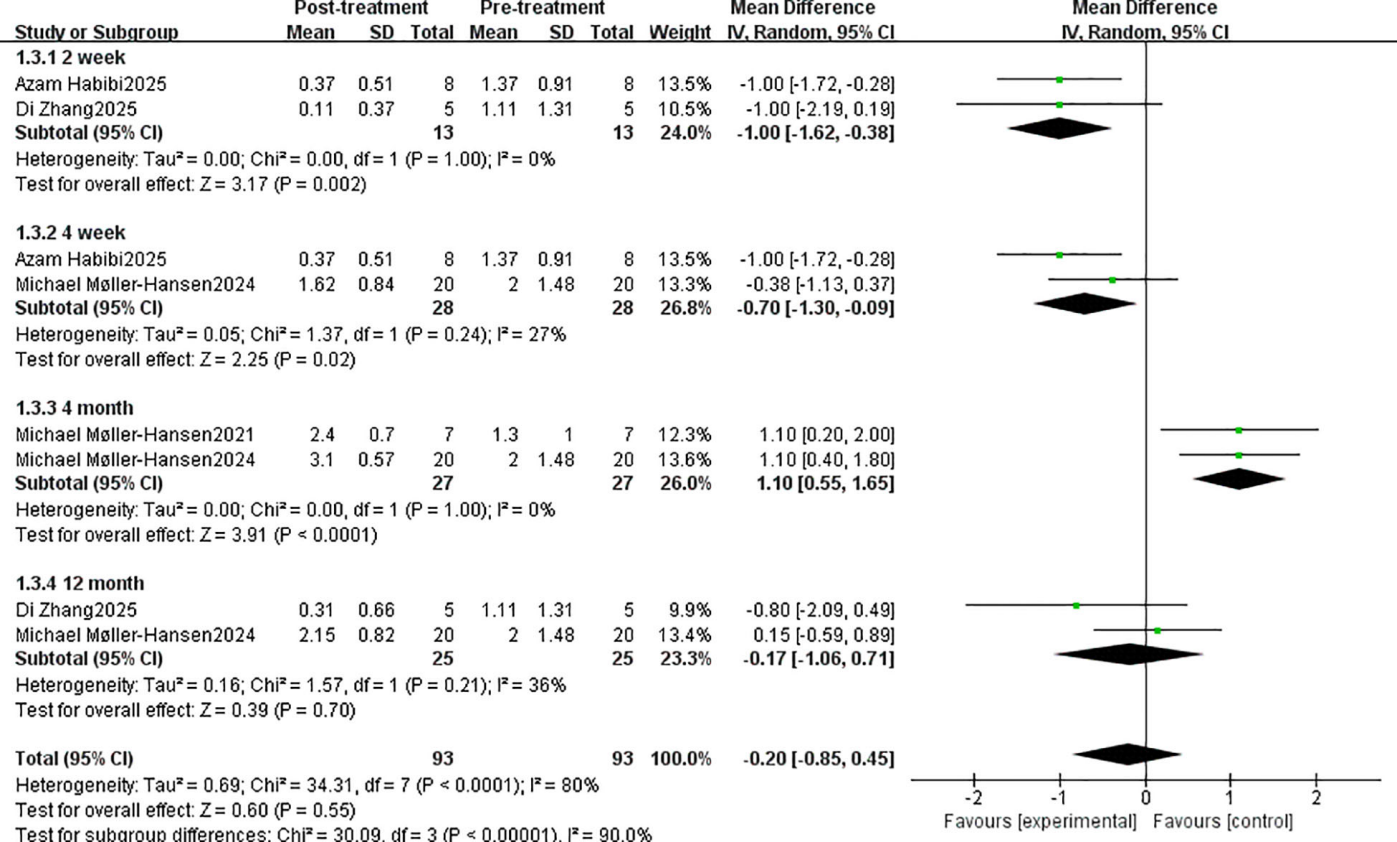
Oxford scoring system for assessing the efficacy of stem cell therapy.

The Oxford score showed a non-significant change at -0.20 (95% CI: -0.85, 0.45; P = 0.55). The MD at 2 weeks, 4 weeks, 4 months, and 12 months was -1.00 (95% CI: -1.62, -0.38; P < 0.002), -0.70 (95% CI: -1.30 to -0.09, P < 0.02), 1.10 (95% CI: 0.55 to 1.65, P < 0.0001), and -0.17 (95% CI: -1.06 to 0.71, P < 0.70) at 2 weeks, 4 weeks, 4 months, and 12 months, respectively (**Figure 4**). Statistically significant differences were observed at all time points except 12 months post-treatment. Although statistically significant at 4 months, the Oxford classification showed an increasing trend.

**Figure 5:**
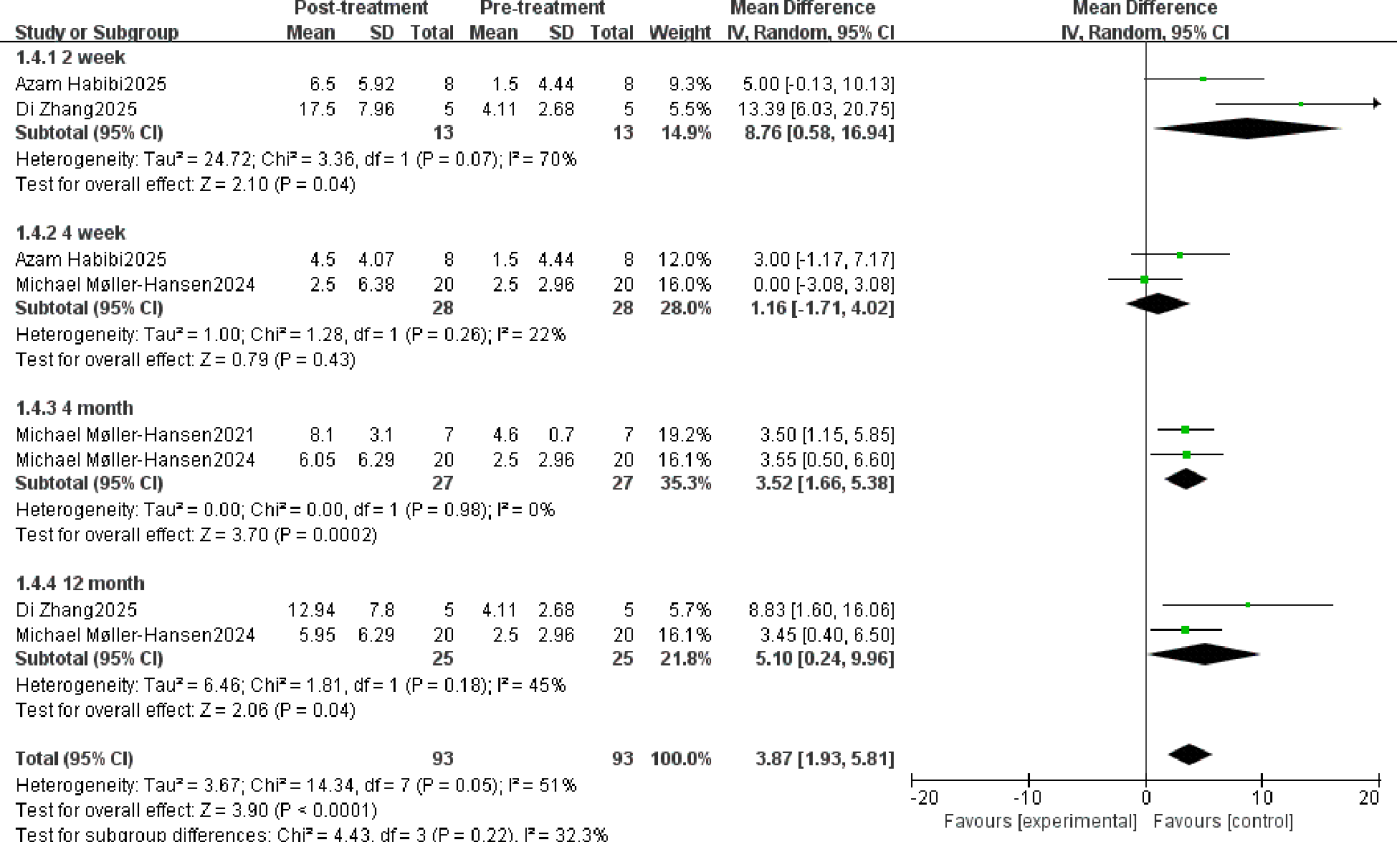
The Schirmer test evaluates the efficacy of stem cell therapy.

Schirmer test score change: 3.87 (95% CI: 1.93, 5.81; P < 0.0001). The MD at 2 weeks, 4 weeks, 4 months, and 12 months was 8.76 (95% CI: 0.58, 16.94; P < 0.04), 1.16 (95% CI: -1.71, 4.02; P < 0.43), 3.52 (95% CI: 1.66, 5.38, P < 0.002), and 5.10 (95% CI: 0.24, 9.96, P < 0.04) at 2weeks, 4 weeks, 4 months, and 12 months respectively (**Figure 5**). Results indicate statistically significant differences in Schirmer test scores following stem cell therapy at all time points except 4 weeks post-treatment.

**Figure 6:**
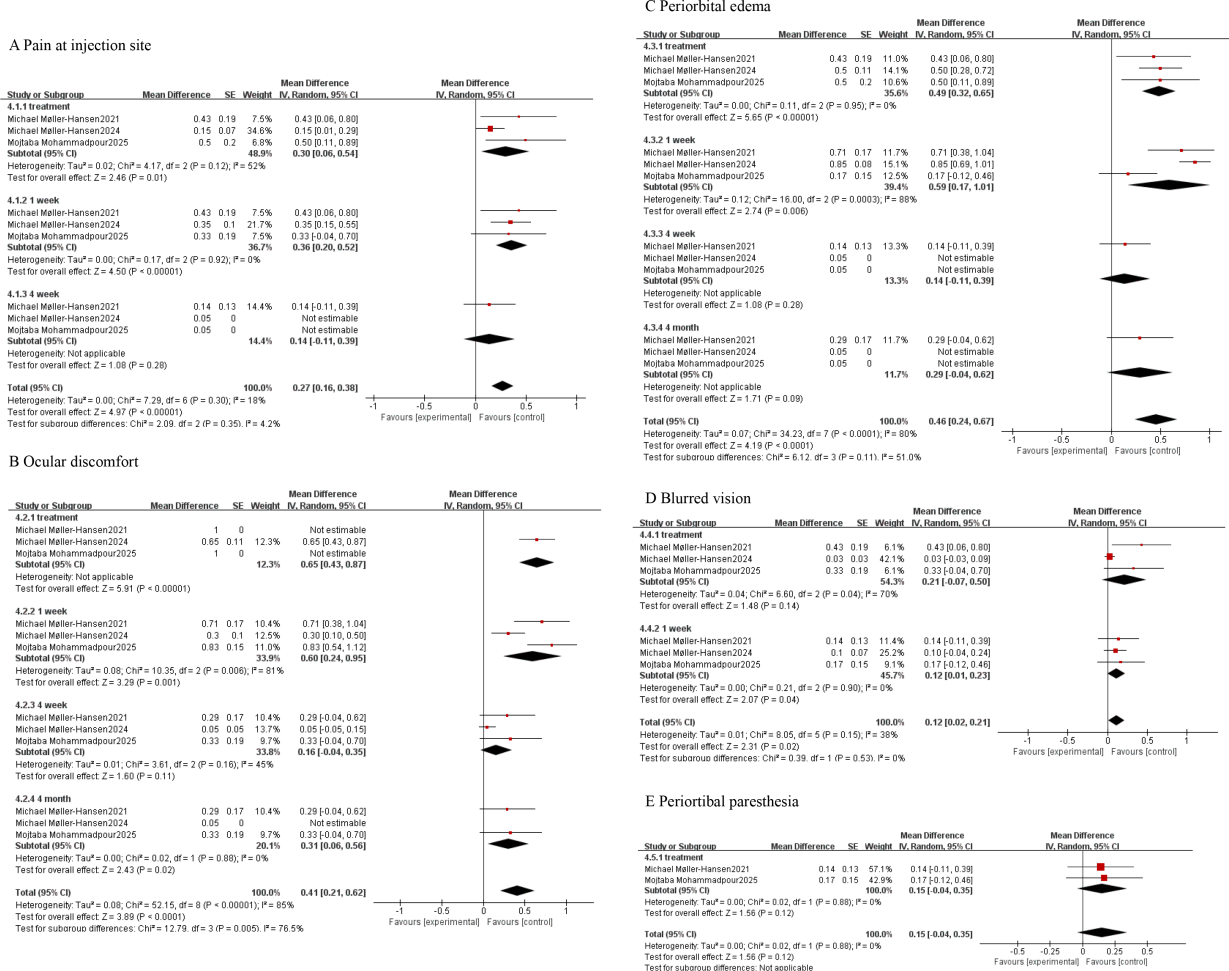
Subgroup analysis of adverse events occurring in the eye following stem cell therapy. **(A)** Pain at injection site. **(B)** Ocular discomfort. **(C)** Periorbital edema. **(D)** Blurred vision. **(E)** Periortibal paresthesia.

Pooled analysis revealed the incidence of adverse events by single-group rate. Subgroup analysis indicated an injection pain incidence of 14% at 4 weeks (95% CI: -11%, 39%, P = 0.28) (**Figure 6A**). Ocular discomfort occurred in 16% of cases at 4 weeks (95% CI: -4%, 35%, P = 0.11) (**Figure 6B**). Periorbital oedema occurred in 14% of cases at 4 weeks (95% CI: -11%, 39%, P = 0.28) (**Figure 6C**). The incidence rates of blurred vision and periorbital paresthesia during treatment were 21% (95% CI: -7%, 50%, P = 0.14) (**Figure 6D**) and 15% (95% CI: -4%, 35%, P = 0.12) (**Figure 6E**), respectively, with no statistically significant differences. All outcomes are summarised in (**Table 2**, **Table 3**). No statistically significant differences in adverse events were observed at longer follow-up periods or across individual study endpoints.

**Table 2:** Summary of quantitative analysis and heterogeneity analysis for outcome indicator.

Outcome indicator	Subgroup analysis	Post-treatment vs pre-treatment			
		MD	95%CI	Z value	P value
OSDI score	2 week	-14.58	-21.22,-7.95	4.31	<.0001
	4 week	-12.89	-19.49,-6.29	3.83	.0001
	4 month	-19.77	-28.16,-11.39	4.62	<.00001
	12month	-16.20	-24.91,-7.50	3.65	.0003
NIKBUT first	2 week	3.48	1.80,5.16	4.05	<.0001
	4 week	3.66	-1.51,8.82	1.39	.17
	4 month	3.70	2.13,5.26	4.63	<.00001
	12 month	2.70	0.27,5.13	2.18	.03
Oxford score	2 week	-1.00	-1.62,-0.38	3.17	.002
	4 week	-0.70	-1.30,-0.09	2.25	.02
	4 month	1.10	0.55,1.65	3.91	<.0001
	12 month	-0.17	-1.06,0.71	0.39	.70
Schirmer test score	2 week	8.76	0.58,16.94	2.10	.04
	4 week	1.16	-1.71,4.02	0.79	.43
	4 month	3.52	1.66,5.38	3.70	.0002
	12 month	5.10	0.24,9.96	2.06	.04

**Table 3:** Merger effect and heterogeneity of safety outcome measures.

Safety outcome measures	Subgroup analysis	Merger effect			
		Rate	95%CI	Z value	P value
Pain at injection site	treatment	0.30	0.06,0.54	2.46	.01
	1 week	0.36	0.20,0.52	4.50	<.00001
	4 week	0.14	-0.11,0.39	1.08	.28
Ocular discomfort	treatment	0.65	0.43,0.87	5.91	<.00001
	1 week	0.60	0.24,0.95	3.29	.001
	4 week	0.16	-0.04,0.35	1.60	.11
	4 month	0.31	0.06,0.56	2.43	.02
Periorbital edema	treatment	0.49	0.32,0.65	5.65	<.00001
	1 week	0.59	0.17,1.01	2.74	.006
	4 week	0.14	-0.11,0.39	1.08	.28
	4 month	0.29	-0.04,0.62	1.71	.09
Blurred vision	treatment	0.21	-0.07,0.50	1.48	.14
	1 week	0.12	0.01,0.23	2.07	.04
Periorbital paresthesia	treatment	0.15	-0.04,0.35	1.56	.12

## Discussion

### Statement of key findings

We conducted a meta-analysis based on five studies to evaluate the efficacy and safety of stem cell therapy for dry eye syndrome caused by Sjögren’s syndrome. Results confirmed that stem cells demonstrated significant therapeutic effects compared to pre-treatment levels. In summary, stem cell therapy resulted in an average reduction of 15.1 points in OSDI scores, an average increase of 3.26 seconds in NIKBUT first post-treatment, a significant decrease of 0.20 in Oxford scores post-treatment, and an average increase of 3.87 mm in Schirmer test scores. These findings indicate the potential of stem cell therapy for dry eye syndrome. However, single-group incidence analysis of adverse events post-treatment revealed statistically significant short-term and one-week occurrences of pain at the injection site, ocular discomfort, and periorbital oedema. These symptoms largely resolved by four weeks. Blurred vision and periorbital paresthesia were reported in isolated cases but showed no statistical significance either in the short or long term.

Although the pooled analysis did not show a statistically significant increase in adverse events, this should not be interpreted as evidence of no clinical risk. The relatively high point estimate and wide confidence interval suggest substantial uncertainty, likely related to the small sample size. Clinically, the reported adverse events were mostly local and non-serious, indicating acceptable short-term tolerability; however, a potential increase in risk cannot be excluded. Some adverse events, such as pain at the injection site and periorbital oedema, may be more closely related to the administration technique or the local injection process, rather than necessarily indicating biological toxicity of the stem cells themselves. The adverse events reported to date are predominantly localised, short-term and reversible; therefore, the safety findings should be interpreted in conjunction with procedural factors. Consequently, concerns regarding stem cell therapy arising in the short term—including at the time of treatment and 1week post-treatment—may limit its acceptability in some patients and underscore the need for enhanced monitoring during the therapeutic process.

### Strengths and weaknesses

This review represents the first systematic evaluation and meta-analysis synthesising clinical trial evidence on the efficacy and safety of stem cell therapy for dry eye syndrome in Sjögren’s syndrome. Furthermore, the study comprehensively covered multiple efficacy measures, including OSDI scores, NIKBUT first, Oxford scores, and Schirmer test scores, thereby presenting a holistic picture of stem cell therapeutic characteristics. It provides a nuanced perspective on the safety profile of stem cell therapy across different adverse events. Analyses at varying follow-up time points enable detailed characterisation of the safety features of stem cell-derived medications. However, the study has limitations. To date, only five clinical trials have been conducted in this patient population. Data at 1 week and 4 weeks were missing in the 2021 study by Michael Møller-Hansen et al. Mojtaba Mohammadpour et al. (2025) published their findings as a preprint, yet data for 1 week, 4 weeks, and 16 weeks were absent. This data deficiency may impact overall study conclusions. The relatively small number of included trials potentially limits analytical breadth; publication bias could not be assessed due to the limited study volume; and methodological variations between trials precluded analysis of dose-dependent effects of stem cell therapy. The reliability of the subgroup analyses may be limited by variations in follow-up duration across studies and missing data at some time points, which reduced the number of studies included in each subgroup and may have lowered the precision and stability of the time-specific estimates.

Because OSDI is a subjective patient-reported outcome, the observed improvement may have been influenced to some extent by placebo effects, patient expectations, or other non-specific factors, particularly in self-controlled or non-blinded studies. Although the inclusion of double-blind randomized clinical trials and the concurrent improvement in some objective parameters partially support the credibility of the findings, the OSDI results should nevertheless be interpreted with caution.

An additional limitation should be acknowledged regarding the inclusion of both randomized controlled trials and self-controlled studies. Although all studies were analyzed using a unified framework based on within-group change from baseline, these designs differ inherently in their susceptibility to bias, particularly with respect to confounding, placebo effects, and regression to the mean. Therefore, while this approach enabled us to synthesize the limited available clinical evidence in this field, the pooled estimates should be interpreted cautiously. Future meta-analyses with a larger evidence base should ideally perform design-specific analyses or sensitivity analyses restricted to randomized controlled trials.

A further limitation is the very small evidence base currently available for this topic. Only five clinical studies involving a total of 114 participants were included in this meta-analysis, which substantially limits the precision and generalizability of the findings. In addition, missing data at several follow-up time points, variation in study design, and the inclusion of preprint data may further reduce confidence in the results. Accordingly, although the present analysis provides encouraging preliminary evidence that stem cell therapy may improve dry eye-related outcomes in patients with Sjögren’s syndrome, these findings should be regarded as exploratory rather than definitive. Future larger, rigorously designed, and preferably multicenter randomized controlled trials are warranted to confirm the efficacy and safety of this therapeutic approach.

Due to cell products from different sources may have distinct immunomodulatory and paracrine properties, and differences in dose or administration route may alter local exposure, duration of effect, and adverse-event profiles. Therefore, the pooled estimates should be interpreted as average effects across heterogeneous stem cell-based interventions rather than as the effect of a single standardized product or regimen. Given the small number of included studies and participants, we were unable to perform robust subgroup analyses by cell type, dose, or route of administration, and dose-response effects could not be assessed. Future trials should standardize the reporting of cell source, dose, route, and treatment schedule to enable more precise intervention-specific analyses.

### Interpretation and future research

Dry eye syndrome ranks among the most prevalent conditions encountered in ophthalmic outpatient settings (21, 22), with a global prevalence ranging from 5% to 50%. The patient population predominantly comprises middle-aged and elderly women. Sjögren’s syndrome constitutes the primary cause of aqueous deficiency dry eye globally, affecting 0.1% to 0.72% of the worldwide population (23–25). The most common conventional treatment for dry eye syndrome is artificial tear eye drops. Artificial tears provide only short-term relief and are effective primarily for mild dry eye. For moderate to severe dry eye, punctal occlusion offers good results but may exacerbate ocular surface damage (26, 27). Treatment challenges persist for Sjögren’s syndrome patients, yet stem cells have opened new therapeutic avenues, demonstrating promising prospects in recent studies.

Numerous animal studies have demonstrated the efficacy of stem cells in treating dry eye syndrome, supported by multiple qualitative reviews (28, 29). The therapeutic potential of SC-Exos in managing severe DED relies on their capacity to selectively deliver carried substances directly to ocular infiltrating immune cells and endothelial cells, thereby modulating their phenotype and function. Immunomodulatory proteins and microRNAs derived from MSC-Exos may induce ocular infiltrating macrophages, dendritic cells (DCs), and T cells to transition towards anti-inflammatory and immunosuppressive phenotypes, thereby alleviating persistent inflammation (30, 31). This study indicates that although preclinical experiments demonstrate promising results, certain challenges persist. There is a lack of standardised protocols for stem cell isolation and characterisation, with variations in purity, particle size, and contents observed across different isolation methods.

A series of clinical trials evaluating stem cells aims to assess their efficacy and safety for dry eye syndrome. Michael Møller-Hansen’s 2024 study examined outcomes at 12-month follow-up after ASC injection, with a 16.1-point reduction in OSDI scores at final follow-up. Azam Habibi’s 2025 study assessed outcomes at 3-month follow-up after MSC instillation, showing a 7.5-point decrease in OSDI scores at final follow-up. Di Zhang’s 2025 study examined outcomes over a 12-month follow-up period after MSC instillation, with the final follow-up OSDI score decreasing by 16.2 points. Michael Møller-Hansen’s 2021 study assessed outcomes over a 4-month follow-up period after ASC injection, with the final follow-up OSDI score decreasing by 24.8 points. Mojtaba Mohammadpour 2025 study aimed to observe outcomes during a 6-month follow-up period after ASC injection, with a final follow-up OSDI score reduction of 20.54 points. Each study contributes to a comprehensive understanding of stem cell therapy’s potential as a transformative treatment for Sjögren’s syndrome.

Despite the potential benefits of stem cell therapy, the occurrence of adverse events remains a concern in the treatment of dry eye syndrome. Higher rates of adverse events may influence patient choice and must be weighed against therapeutic benefits. In clinical trials, the primary adverse events reported were pain at the injection site, ocular discomfort, periorbital oedema, blurred vision, and periorbital paraesthesia. Our findings indicate that these adverse events largely subsided by week 4 of treatment. Although some complications rebounded during longer follow-up periods, the overall pattern remained largely unchanged. Additionally, we observed an increased risk of injection site reactions. Complications associated with the injection procedure may also potentially reduce patient compliance. Therefore, the safety profile of stem cell therapy must be thoroughly considered. Although most reported adverse events were transient and self-limiting, their relatively frequent occurrence may still be clinically relevant in terms of patient comfort and treatment acceptability. Therefore, safety should not be judged solely on statistical significance, especially in small studies with limited power to detect uncommon or delayed events. Some local adverse events, such as injection-site pain and periorbital edema, are likely to be related at least in part to the injection procedure itself rather than the stem cell product alone. However, because the included studies did not consistently separate procedure-related from product-related events, a definitive attribution cannot be made.

From a clinical perspective, stem cell therapy may offer benefits beyond symptom relief through anti-inflammatory and immunomodulatory effects, showing potential for patients with severe or refractory SS-DED. However, despite the favorable improvements observed in this meta-analysis, there is still insufficient high-quality head-to-head evidence comparing stem cell therapy with established treatments such as cyclosporine or other immunomodulatory agents. Therefore, its comparative therapeutic value remains uncertain, and stem cell therapy should currently be considered a promising complementary or alternative strategy rather than a proven replacement for existing standard treatments.

Future studies should directly compare stem cell therapeutics with alternative treatments in this population, while thoroughly examining adverse events to comprehensively and robustly evaluate efficacy and safety. Furthermore, strategies to enhance tolerability of stem cell therapy may improve its clinical utility. These include strengthening patient education, adopting standardised stem cell preparation and transport protocols (32, 33) to minimise adverse reactions, and exploring novel therapies with comparable or superior efficacy and safety profiles.

Although the present analysis primarily focused on dry eye syndrome associated with Sjögren’s syndrome, and most currently available evidence is derived from patients with primary Sjögren’s syndrome, our findings may have limited implications for secondary Sjögren’s syndrome as well. Primary and secondary Sjögren’s syndrome share several key immunopathological features, including lymphocytic infiltration of the lacrimal glands, chronic ocular surface inflammation, tear film instability, and immune-mediated impairment of exocrine secretion (34). On this basis, the therapeutic mechanisms of stem cell-derived medicinal products—particularly their immunomodulatory, anti-inflammatory, and tissue-reparative effects—could theoretically also confer benefit in secondary Sjögren’s syndrome-associated dry eye. However, such extrapolation should be made cautiously. In secondary Sjögren’s syndrome, the presence of an underlying systemic autoimmune disease, such as rheumatoid arthritis or systemic lupus erythematosus, may alter the inflammatory milieu, disease severity, concomitant medications, and ultimately treatment responsiveness. Therefore, although our results suggest a potential broader translational relevance, they should not be interpreted as direct evidence for secondary Sjögren’s syndrome. Future well-designed studies should specifically include or stratify patients with secondary Sjögren’s syndrome to determine whether the efficacy and safety profile of stem cell-derived therapies differs across Sjögren’s syndrome subtypes.

This meta-analysis holds significant importance as the first systematic and comprehensive evaluation of the safety and efficacy of stem cell-derived drugs in alleviating ocular dryness and associated symptoms, further highlighting its unique contribution to the existing literature. Future research still requires high-quality randomised controlled trials to validate these conclusions.

## Conclusion

This meta-analysis indicates that stem cell-derived medicinal products effectively reduce OSDI scores, increase tear production, and enhance tear film stability in dry eye syndrome caused by Sjögren’s syndrome. However, the elevated incidence of adverse events in the short term suggests that benefits and risks must be carefully weighed prior to clinical application, with rigorous monitoring and close follow-up required during treatment. This study underscores the necessity for further research directly comparing stem cell-derived drugs with other therapeutic agents to comprehensively elucidate their therapeutic value in dry eye syndrome associated with Sjögren’s syndrome. These findings should be interpreted cautiously, as the pooled estimates reflect average effects across heterogeneous stem cell-based interventions rather than a single standardized therapeutic regimen.

## Data Availability

The original contributions presented in the study are included in the article/**Supplementary Material**. Further inquiries can be directed to the corresponding author.
