# Supplementary material for: Efficacy and safety of stem cell therapy for dry eye syndrome in Sjögren’s syndrome: a systematic review and meta-analysis
Source: Front Immunol. 2026 Apr 29;17:1834453. doi: 10.3389/fimmu.2026.1834453 (PMC13182517; doi:10.3389/fimmu.2026.1834453)
Supplement: Supplementary file 1 [file Table1.docx]

Supplement figure1 Risk of bias graph for RCT


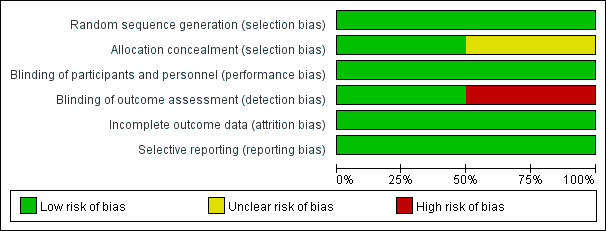


Supplement figure2 Risk of bias summary for RCT


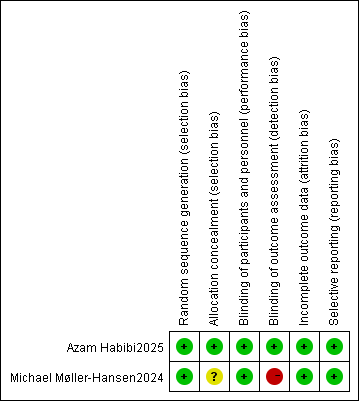


Supplement table1

| **MINORS** | Definition of objective | Inclusion of patient coherence | Expected data collection | Accuracy of endpoints | Objectivity of endpoints | Adequacy of  follow-up | Few loss to follow-up rate | Estimation of sample size | **Total score** |
| --- | --- | --- | --- | --- | --- | --- | --- | --- | --- |
| Di Zhang  2025 | 2 | 1 | 2 | 2 | 2 | 2 | 2 | 1 | 14 |
| Michael Møller-Hansen  2021 | 2 | 1 | 2 | 2 | 2 | 1 | 2 | 1 | 13 |
| Mojtaba Mohammadpour2025 | 2 | 1 | 2 | 2 | 2 | 2 | 2 | 1 | 14 |

Risk of bias the Non-randomized controlled trial included.

Search strategy

Pubmed:

| Query | Results |
| --- | --- |
| Search: (Stem cells or Mesenchymal stem cells or Adipose tissue or Adipose-derived) AND (Dry Eye Syndrome or Dry Eye Disease) AND (Random or RCT or RCTs or ‘randomised controlled trial’ or trial* or ‘clinical trial’) | 66 |

Embase:

| No | Query | Results | Date |
| --- | --- | --- | --- |
| #1 | ('stem cells'/exp OR 'stem cells' OR 'mesenchymal stem cells' OR 'adipose tissue' OR 'adipose-derived') AND ('dry eye syndrome' OR 'dry eye disease')AND ('random' OR 'rct' OR  'rcts' OR 'randomised controlled trial' OR 'trial*' OR 'clinical trial') | 53 | 16-Jan-25 |

Cochrane library:

21 Trials matching (Stem cells or Mesenchymal stem cells or Adipose tissue or Adipose-derived) AND (Dry Eye Syndrome or Dry Eye Disease) AND (Random or RCT or RCTs or ‘randomised controlled trial’ or trial* or ‘clinical trial’) in Title Abstract Keyword - (Word variations have been searched)
